# A cross-sectional study of the relationship between parents’ and children’s physical activity

**DOI:** 10.1186/s12889-016-3793-3

**Published:** 2016-10-28

**Authors:** Jodie A. Stearns, Ryan Rhodes, Geoff D. C. Ball, Normand Boule, Paul J. Veugelers, Nicoleta Cutumisu, John C. Spence

**Affiliations:** 1 Faculty of Physical Education and Recreation, University of Alberta, 1-155 Van Vliet Complex, AB T6G 2H9 Edmonton, Canada; 2 School of Exercise Science and Physical Health Education, University of Victoria, Victoria, Canada; 3 Department of Pediatrics, Faculty of Medicine & Dentistry, University of Alberta, Edmonton, Canada; 4 Department of Public Health Sciences, University of Alberta, Edmonton, Canada; 5 Institut national de la recherche scientifique- Institut Armand-Frappier, Unité d’épidémiologie et biostatistique/Unit of Epidemiology and Biostatistics, Laval, Canada

**Keywords:** Children, Parents, Physical activity, Pedometers, Questionnaires

## Abstract

**Background:**

Though parents’ physical activity (PA) is thought to be a predictor of children’s PA, findings have been mixed. The purpose of this study was to examine the relationship between pedometer-measured steps/day of parents’ and their children and potential moderators of this relationship. We also assessed the parent–child PA relationship as measured by questionnaires.

**Methods:**

Six-hundred and twelve 7–8 year olds and one of their parents wore *Steps Count (SC)-T2* pedometers for four consecutive days. Parents reported their PA from the last seven days and their child’s usual PA. Hierarchical linear regressions were used to assess the parent–child PA relationships, controlling for covariates. Gender (parent, child), gender homogeneity, weight status (parent, child), weight status homogeneity, and socioeconomic status (SES) variables (parent education, household income, area-level SES) were tested as potential moderators of this relationship. Partial *r*’s were used as an estimate of effect size.

**Results:**

Parents’ steps was significantly related to children’s steps (*r*_partial_ = .24). For every 1,000 step increase in parents’ steps, the children took 260 additional steps. None of the tested interactions were found to moderate this relationship. Using questionnaires, a relatively smaller parent–child PA relationship was found (*r*_partial_ = .14).

**Conclusion:**

Physically active parents tend to have physically active children. Interventions designed to get children moving more throughout the day could benefit from including a parent component. Future research should explore the mechanisms by which parents influence their children, and other parent attributes and styles as potential moderators.

## Background

Identifying the primary determinants of children’s physical activity (PA), is an important public health issue [1]. Undoubtedly, parents play a key role in the development of their children’s health behaviors. For instance, the Integrated Model of Physical Activity Parenting (IMPAP) describes how parents’ attributes and parenting practices influence their children’s PA attributes and outcomes [2]. The literature to date has focused largely on two parent attributes: parental support for PA and parental PA (often described as parental modeling of PA). A recent meta-analysis [3] found that parental support was moderately related to children’s PA (*r* = .38). Parental PA, on the other hand, was a weak predictor of child PA (*r* = .16). Several previous reviews have also reported mixed findings across studies for parental PA [4, 5]. This is surprising considering parents are routinely encouraged to be active role models for their children by health professionals [6].

Several potential reasons for the discrepancy across studies have been proposed including the over reliance on subjective measures of PA (i.e., parental-proxy or self-report questionnaires) which may bias results [6, 7], differences according to the intensity of PA (e.g., moderate-to-vigorous PA [MVPA], total PA) [8], and parents having less influence as children become older and gain independence [3, 9]. Parental influence could also vary by within and between family factors such as parent and child gender, and socioeconomic status (SES).

In support of the importance of using objective measures, the meta-analysis by Yao and Rhodes found a trend towards studies using objective assessments of youths’ PA (*r* = .24) having a stronger parent–child PA relationship compared to those using subjective measures (*r* = .13) [3]. Heterogneity was also present, particularly in the questionnaire-based studies, suggesting that questionnaires tend to introduce measurement error that attenuates effects. Comparing the parent–child PA relationship using objective and subjective measures in the same sample would provide evidence to test this assumption.

Several studies have examined the relationship between parents’ and their children’s objectively measured steps/day [10–14], accelerometer-measured counts [9, 15–17], light PA [18], and/or MVPA [9, 17, 19]. Most studies have reported a significant relationship with at least one parent, however many have also observed differences by parent or child gender. For example, Jacobi et al. [10] observed a mother effect whereby mother’s steps (but not the father’s steps) were correlated to their offspring’s steps in a sample of French nuclear families. In contrast, significant relationships between steps/day in father-sons, father-daughters, and mother-sons were found in a representative sample of Canadian children and their parents, yet the mother-daughters PA relationship did not reach statistical significance (*p* = .08) [8]. Thus it is unclear from the existing literature whether the parent–child PA relationships differs by child or parent gender. Studies that formally test whether gender specific relationships are significantly different from one another could provide some clarity.

Total PA performed throughout the day, as well as higher intensity activity, are important for the health of young people [20], and thus it is important to understand the correlates and determinants of both of these outcomes. Pedometers are a reliable and valid measure total ambulatory activity performed throughout the day [21–23]. They are also affordable and accessible to practitioners and families, making the findings of pedometer studies easy to apply to real-life settings [8]. As mentioned, several studies have assessed the parent–child PA relationship using pedometers. For example, 10 additional minutes of parental MVPA resulted in one additional minute of MVPA in 1267 children aged 5–6 years in the UK [24]. Similarly, a 2500 step/day increase in parent’s steps resulted in 752–1143 step increase in a sample of Czech children aged 4–7 years [11].

As illustrated in the IMPAP, the influence of parental variables on children’s PA can vary depending on different child and parent attributes. Examining moderators (e.g., education, income) is important because it can provide information about whom and under what circumstances specific interventions may be effective [2, 25]. Thus, testing a number of potential moderators of the parent–child PA relationship could provide important insights into whether family-based interventions will be effective for everyone or more effective for specific subgroups. Disentangling potential moderators of this relationship could also shed some light on the mixed findings in the literature. SES may be an important moderator to consider as lower SES families often have less access to quality facilities and less time and resources to be active [26–28]. For example, with 286 nuclear families including children aged 8–18 years, Jacobi et al. [10] found significant mother-offspring step count correlations with employed mothers (ICC = .24) and non-significant correlations with unemployed mothers (ICC = .11), however the difference was not formally tested. Considering overweight/obese children and adults are often found to be less active than those who are non-overweight [20, 29, 30], the body size of the children and parents could impact the parent–child PA relationship. It is also possible that children may relate to and imitate their parent(s) if they are of a similar body size, indicating a “weight status homogeneity effect”.

The purpose of our study was to examine the relationship between parents’ and children’s PA in a sample of 7- to 8-year-olds and one of their parents. Research question 1 examined the relationship between pedometer-measured steps/day in parents and their children. Research question 2 examined whether gender (parent, child), gender homogeneity, weight status (parent, child), weight status homogeneity, and SES variables (parent education, household income, area-level SES) moderated this relationship. Research question 3 examined the parent–child PA relationship as measured by questionnaires. Consistent with Yao and Rhodes [3], we hypothesized a small but positive relationship would exist between PA levels in parents and their children. We also hypothesized a stronger parent-child PA relationship with pedometer-determined compared to questionnaire-determined PA. Due to the inconsistencies in the existing literature, the moderation analyses were exploratory.

## Methods

### Participants and procedures

This is a cross-sectional study of 7–8 year old children and parents living in Edmonton, Alberta, Canada (population: ~1,000,000) and the greater metropolitan area. The children were a part of the SHAPEs of Things to Come study, a longitudinal follow-up to the Spatial Health Assessment of Physical Environments (SHAPEs) study [31–34]. In SHAPEs (November 2005 to August 2007), parents and children (~4-5 years) were recruited at preschool immunization appointments at local community health centers where ~75 % of Edmonton-area children receive their preschool immunizations. Of the 1,715 children who participated in SHAPEs, parents of 1,377 children (80.3 %) agreed to be re-contacted for future research. Parents were contacted about the current study between April 2009 and March 2011. Overall, 668 parent–child dyads agreed to participate, representing a 39.0 % response rate.

Of the children who participated, 92 % were 7- or 8-years-old. Because the parent–child PA relationship may differ by age we decided to use only the 7–8 year olds in this analysis, leaving a final sample size of 612 parent–child dyads (53 % girls, 84 % mothers). In this sample, 76 % of the parents had finished university or college, and 78 % of the families had a household income > $80,000/year. Using data from the baseline study (SHAPEs), those who were included in the current study did not significantly differ on age, unemployment rate, or parent-proxy reported PA from those who did not participate. Participants in our study did have significantly lower BMI’s, and lived in areas with higher household income and education levels; however the effects were small (*d* = .15–.23).

Eligible parents were sent an information letter, consent form, and a brief questionnaire in the mail. Interested families attended appointments at an off-campus athletic fitness facility where procedures were explained and informed assent and consent were obtained from children and parents, respectively. Anthropometric assessments were completed with children while parents completed an additional questionnaire. At the end of their visit, families were provided with unsealed pedometers and instructed to wear the device for four consecutive days, including three weekdays and one weekend day (i.e., either Sunday to Wednesday or Wednesday to Saturday). Four days of monitoring was chosen because the literature at the time deemed this as an adequate period for determining habitual PA in children [21, 35, 36]. They were also instructed to record their steps in a logbook each night and to reset the counter for the next day. The pedometers were worn on the belt or waistband in the right mid-line of the thigh, which is the most accurate position for pedometers [37]. For convenience, families were allowed to choose which parent attended the appointment and wore the pedometer (of those that attended the appointment, 15 % were fathers and 83 % mothers; of those who returned their pedometers, 16 % were fathers and 84 % were mothers). Once the pedometer-recording period was over, parents mailed the pedometers and logbooks back to the researchers using a preaddressed, postage-paid envelope. Of the 612 parent–child dyads who completed the initial assessments, 440 returned their pedometers and logbooks. Families received modest tokens of appreciation (value: ~$20) for participation. The study was approved by the University of Alberta’s Ethics Board.

### Measures

#### Pedometers

Unsealed *Steps Count (SC)-T2* steps and activity time pedometers (Steps Count, Deep River, ON) were used to measure PA in children and parents. This pedometer, which is mechanically identical to the Walk for Life pedometer, has demonstrated good reliability and accuracy in adults [38] and children 5–11 years-old [39]. The SC-T2 pedometers have a 3-s delay function to reduce erroneous steps due to jostling. Participants that indicated the pedometer was not registering steps properly were marked as missing for the entire four days. Those that indicated they forgot to put the pedometer on for a significant period of time (i.e., more than 3 h) were flagged and that day of measurement was marked as missing. Most participants (90 %) wore the pedometers for four consecutive days and for three weekdays and one weekend day (62 %) as instructed. Average steps were calculated as the mean across the measured days.

#### Physical activity questionnaires

Parents reported their children’s PA using the CLASS survey, which has shown acceptable psychometric properties in 10–12 year olds [40]. Parents indicated how often (frequency per week) and for how long (in minutes) their child usually does common activities in the current season. Because this survey was developed in Australia, the activities and wording were modified to be relevant to a Canadian context and included swimming, soccer, ballet/dance, gymnastics, skating, hockey, bike riding, gym activities, active play, “other”. Average PA in min/day was calculated for entire week. The modified version of this questionnaire was used in the first study involving these participants (SHAPEs) [31, 32]. The Godin Leisure Time Exercise Questionnaire [41] was used to measure parents’ leisure-time MVPA. Parents reported how often (frequency per week) they do strenuous and moderate exercise for more than 15 min during their free time. To obtain a total metabolic equivalent (MET) score, the strenuous score was multiplied by 9, the moderate score was multiplied by 5, and these two scores were summed. To be consistent with the steps/day measure, average METS/day was calculated. This tool is well-established and has demonstrated validity and reliability data [42].

#### Anthropometry

Weight and height were measured twice and recorded to the nearest .1 kg and .1 cm, respectively. If a discrepancy existed between the two measurements (>.5 cm for height; >.2 kg for weight), a third measurement was taken and the three measurements were averaged. Children’s sex and age specific body mass index (BMI) z-scores were calculated using the World Health Organization’s (WHO) growth reference [43]. The BMI *z*-score was used to categorize children as *non-overweight* (≤1), *overweight/obese* (≥1.01). Parental height and weight were self-reported, which was used subsequently to calculate BMI and group individuals as *non-overweight* (<25 kg/m^2^), *overweight/obese* (≥25.01 kg/m^2^) [44]. For both the children and parents, the *non-overweight* category included participants who could be classified as “thin” (children *n* = 87) or “underweight” (parents *n* = 10) or “healthy”. A weight status homogeneity variable was created by coding parents and children with the same weight status (i.e., both overweight/obese, both non-overweight) as 1 and those with a different weight status as 0.

#### Season

Given the influence of season on PA [31, 45], the season of the assessment day and first complete day of pedometer measurement were calculated. Winter was defined as December to February, spring as March to May, summer as June to August, and autumn/fall as September to November.

#### Demography

Children’s date of birth was used to determine age in years. Parent gender was recorded for the parent who wore the pedometer. Gender homogeneity was created by coding parents and children with the same gender (i.e., both female, both male) as 1 and those with the opposite gender as 0. Household income was categorized into < $80,000 per year and > $80,000 per year for our analyses based on the approximate median income level in Alberta [46]. Parent education was categorized as “has not completed college/university”, “completed college/university”, and “completed graduate degree” for descriptive purposes and “no graduate degree” and “completed graduate degree” for the main analysis.

Area-level SES was calculated using families’ postal code data. GeoPinPoint™ Suite software [47] was used to locate the family address into its corresponding dissemination area, which is defined as one or more adjacent blocks of 400–700 people [48] around the home. Based on the 2006 Canadian Census [49], the proportion of people with low education were subtracted from the proportion of people with high education in each dissemination area.

### Analysis

All analyses were conducted using IBM SPSS Statistics version 23. Outliers for the pedometer data were identified as days with <1,000 or >30,000 steps/day for children and <1,000 or >25,000 for adults were set as missing [22]. Outliers for the child PA questionnaire (>6 h/day), and for the other continuous variables (≥ ± 3.29 SD) were truncated [50].

Of the 28 variables included in this study, 83 % were missing on at least one value (number of non-missing values for each variable is available in Table 1). Across cases/participants, 55 % were missing on at least one variable, and across the entire dataset, 13 % of the values were missing. Missing and non-missing cases were compared for variables with >10 % missing data. Significant (*p* < .05) or marginally significant (*p* < .10) differences existed on parental BMI for parents’ and children’s steps/day. Importantly, families who participated in the initial assessment and those that returned the pedometers did not differ on parent self-reported leisure time MVPA (*t* = −.67, *p* = .50) or children’s parental-proxy reported PA (*t* = −.38, *p* = .38). We therefore assumed at least a partial missing at random mechanism and imputed all of the missing data (including all covariates, predictor variables, criterion variables) using multiple imputation in SPSS. This procedure uses the fully conditional specification method and imputes data using linear regression for continuous variables and logistic regression for binary variables. We used 100 iterations, which resulted in 100 separate datasets [51]. Relevant variables from the wider dataset (i.e., screen time, aerobic fitness, grip strength, dog ownership, walkability of neighborhood) were included as auxiliary variables.

**Table 1:** Sample characteristics for the non-imputed dataset and the pooled results

	Non-Imputed Dataset		Pooled Result^a^	
	N	Statistic	N	Statistic
Child Characteristics				
Gender – count (%)	612		612	
Boys		289 (47.2)		289 (47.2)
Girls		323 (52.8)		323 (52.8)
Age in years – M (SD)	612		612	
7 years		203 (33.2)		203 (33.2)
8 years		409 (66.8)		409 (66.8)
Weight status – count (%)	658		612	
Non-overweight^b^		472 (78.1)		476 (77.8)
Overweight/obese		132 (21.9)		136 (22.2)
Total steps/day – M (SD)	436	8606 (2860)	612	8558 (3313)
Parental-reported total PA (min/day) – M (SD)	598	107.89 (64.09)	612	107.72 (65.07)
Parent Characteristics				
Parents’ gender – count (%)	612		612	
Fathers		97 (15.8)		97 (15.8)
Mothers		515 (84.2)		515 (84.2)
Weight status – count (%)	550		612	
Non-overweight^b^		309 (56.2)		339 (55.4)
Overweight/obese		241 (43.8)		273 (44.6)
Total steps/day – M (SD)	430	7796 (3088)	612	7741 (2910)
Leisure time MVPA (METS/day) – M (SD)	518	6.91 (4.02)	612	6.96 (4.61)
Socio-economic status indicators				
Parents’ education – count (%)	557		612	
Has not completed college/university		117 (21.0)		145 (23.7)
Completed college/university		382 (68.6)		381 (62.2)
Completed graduate school		58 (10.4)		86 (14.1)
Household Income – count (%)	563		612	
< $80,000/year		114 (20.2)		137 (22.4)
> $80,000/year		449 (79.8)		475 (77.6)
Area-level SES - M (SD)	610	12.12 (19.53)	612	12.07 (19.58)

Note. *PA* physical activity; *MVPA* moderate-to-vigorous physical activity; *SES* socioeconomic status; ^a^the pooled results were estimated using multiple imputation, ^b^the non-overweight category includes thin and healthy weight categories

Pearson product–moment correlations were run to test the unadjusted bivariate relationships between parents’ and children’s PA relationships as measured by pedometers. We also tested this relationship separately by child and parent gender, child and parent weight status, gender homogeneity, weight status homogeneity, parent education, household income, and area-level SES. A Pearson product–moment correlation was also run to test the unadjusted bivariate parent–child PA relationship as measured by questionnaires. Linear regressions were used to test the research questions and partial *r* indicated effect size. Cohen’s [52] recommended effect sizes of small = .10, medium = .30, large = .50 were used to interpret the size of effects. The covariates for all analyzes were child age, gender, and weight status; parent gender, weight status, and education; household income; area-level SES; and season. Each analysis included between 11 and 13 variables. According to the IBM SPSS Statistics SamplePower 3, with 11 covariates (medium combined effect size), one predictor variable (medium effect size) and an interaction term (small effect size), 413 participants were required to detect effects at power = .80 for α = .01. Thus we were sufficiently powered for all analyses.

To address research questions 1 (whether parents’ steps/day was related to children’s steps/day), a linear regression was run with children’s steps/day as the criterion variable and parents’ steps/day and covariates as predictor variables. Coefficients were deemed significant at *p* < .05. To address research question 2 (potential moderators of the parent–child step/day relationship), children’s steps/day was entered as the criterion variable and parent’s steps/day and covariates as predictor variables. One by one we tested potential interactions including parent steps*child gender, parent steps*parent gender, parent steps*gender homogeneity, parent steps*child weight status, parent steps*parent weight status, parent steps*weight status homogeneity, parent steps*parent education, parent steps*household income, parent steps*area SES. In the models where the gender homogeneity and weight status homogeneity interactions were tested, these variables were also included as main effects. Before creating the interaction terms, the continuous variables (i.e., parent steps, area-level SES) were centered on their mean [53]. To control for the increased probability of finding a significant result due to running multiple tests, a more stringent significance level was applied (*p* < .01) to the interactions. For significant or near significant interactions, a simple slopes analysis was performed to determine the beta coefficients and *p-*values for each group. Beta coefficients for the simple slopes were calculated by hand using the pooled results [53]. The pooled results did not provide sufficient information to calculate the significance of the slopes by hand so the *p*-value (set at *p* < .05) was computed using the initial dataset (i.e., before the multiple imputation). To address research question 3 (parent–child PA relationship as measured by questionnaires), children’s proxy-reported PA was entered as the criterion variable and parent self-reported leisure time MVPA and the covariates were entered as predictor variables. Coefficients were deemed significant at *p* < .05.

## Results

Sample characteristics are provided in Table 1. The prevalence of overweight and obesity were 22 % for the children and 45 % for the parents. For the initial assessment participation rates varied by season; 10 % participated in the winter, 14 % in the spring, 48 % in the summer, and 28 % in the fall. The pedometer assessment participation rates also varied by season; 12 % of the parent–child dyads participated in the winter, 15 % in the spring, 44 % in the summer, and 29 % in the fall. Boys (*M* = 9075, *SD* = 4832) took more steps than girls (*M* = 8095, *SD* = 4507), *t*(1339) = 3.65, *p* < .001, *d* = .30. No significant differences existed in steps/day between mothers (*M* = 7773, *SD* = 3136) and fathers (*M* = 7568, *SD* = 7737), *t*(41870) = −.66, *p* = .51, *d* = .07.

### Research question 1: Relationship between parents’ and children’s PA as measured by pedometers

The bivariate, unadjusted Pearson’s correlation between the parents’ and children’s steps was *r* = .25, *p* < .001. The results from the linear regression analysis is presented in Table 2. After controlling for covariates, average parents’ steps predicted children’s steps (*B* = 0.26, *p* < .001), with small to medium sized effects (*r*_partial_ = .24). That is, for every 1,000-step increase in parents’ steps, children took approximately 260 additional steps. The model explained 8.8–15.4 % variance in children’s steps.

**Table 2:** Unstandardized beta coefficients and standard errors from linear regressions predicting children’s physical activity

	Children’s Objectively Measured Physical activity (steps/day)	Children’s Subjectively Measured Physical activity (min/day)
	*B* (SE)	*B* (SE)
Child gender		
Male	ref	ref
Female	−1062.74 (264.64)***	15.97 (36.69)
Parent gender		
Male	ref	ref
Female	132.29 (358.94)	34.98 (51.12)
Child weight status		
Non-overweight	ref	ref
Overweight/obese	−767.69 (320.74)*	0.48 (45.28)
Parent weight status		
Non-overweight	ref	ref
Overweight/obese	−33.29 (300.86)	−35.82 (43.63)
Income		
High (>$80,000/year)	ref	ref
Low (<$80,000/year)	−102.87 (347.89)	2.12 (51.46)
Parent education		
No completion of graduate school	ref	ref
Completed graduate school	−582.35 (425.23)	74.26 (63.28)
Area-level SES	−4.94 (6.99)	−0.84 (1.00)
Season		
Winter	ref	ref
Spring	1246.49 (504.85)*	74.33 (77.63)
Summer	1055.41 (452.62)*	128.22 (66.25)
Fall/autumn	1285.84 (458.90)**	0.01 (69.96)
Parents’ objectively measured PA (steps/day)	.26 (0.6)***	-
Parents’ subjectively measured PA (METS/day)	-	2.18 (.70)**
Adjusted R^2^	.088–.154	018–.052

Note. *p < .05, **p < .01, ***p < .001; *PA* physical activity; *SES* socioeconomic status

### Research question 2: Potential moderators of the parent–child PA relationship as measured by pedometers

Table 3 contains the results from the tests of moderation, along with the bivariate parent-child step correlations separated by levels of the moderators. None of the interactions were significant at the *p* < .01 level. However the interaction between parent steps and income (*B* = .25, *p* = .07, *r*_partial_ = .09), and parent steps and education (*B* = .38, *p* = .02, *r*_partial_ = .11) both approached significance. Specifically, in higher income households (*n* = 475; >$80,000/year) the parent–child PA relationship was significant (*B* = .29, *p* < .001) and in lower income households it was not (*n* = 137, <$80,000/year; *B* = .04, *p* = .98). Further, parents who had completed graduate school (*n* = 86) had a stronger parent–child PA relationship (*B* = .61, *p* < .001) than parents without a graduate degree (*n* = 526, *B* = .23, *p* < .001).

**Table 3:** Tests of potential moderators of the parent–child physical activity relationship as measured by pedometers

Potential moderators	Moderator categories	Bivariate correlations		Test of moderation^a^		
		*n*	*r*	*B* (SE)^a^	*r*_partial_	*p*-value
Child gender	Girls	289	.30***	−0.12 (.09)	-.06	.20
	Boys	323	.23***			
Parent gender	Fathers	97	.27*	0.5 (.11)	.02	.64
	Mothers	515	.25***			
Gender homogeneity^b^	Same gender	326	.22***	−0.07 (.09)	-.04	.44
	Opposite gender	286	.30***			
Child weight status	Non-overweight	476	.24***	.11 (.11)	.04	.31
	Overweight/obese	136	.29**			
Parent weight status	Non-overweight	339	.28***	−0.03 (.10)	-.01	.79
	Overweight/obese	273	.20**			
Weight status homogeneity^c^	Same weight status	372	.30***	0.05 (.10)	.02	.63
	Opposite weight status	240	.17*			
Household income	Low (<$80,000/year)	482	.28***	0.25 (.14)	.09	.07
	High (>$80,000/year)	137	.13			
Parent education	No graduate degree	526	.24***	0.38 (.16)	.11	.02
	Graduate degree	86	.33**			
Area-level SES^d^	Low (< mean)	336	.22***	0.00 (.00)	.07	.14
	High (> mean)	276	.29***			

Note. *p < .05, **p < .01, ***p < .001; SES = socioeconomic status; ^a^potential moderators were tested one by one by adding an interaction term (parent steps*potential moderator) to a linear regression model where child steps was the criterion variable and parent steps, child and parent gender, child and parent weight status, education, household income, and area-level SES were predictor variables; ^b^gender homogeneity was also added to the model as a predictor variable; ^c^weight status homogeneity was also added to the model as a predictor variable; ^d^area-level SES was tested as a continuous variable

### Research Question 3: Relationship between parents’ and children’s physical activity as measured by questionnaires

The bivariate, unadjusted Pearson’s correlation between parents’ and children’s subjectively measured PA was *r* = .15, *p* < .01. The results from the linear regression analysis of the parent–child PA relationship using subjectively measured PA is presented in Table 2. After controlling for covariates, parents’ leisure time MVPA (METS/day) was significantly related to children’s proxy-reported PA (min/day; *B* = 2.18, *p* < .01), with small sized effects (*r*_partial_ = .14). The model accounted for 1.8–5.2 % variance in children’s PA.

## Discussion

The purpose of this study was to examine the relationship between pedometer-measured steps/day of parents and their children, and whether this relationship varied by gender (parent, child), gender homogeneity, weight status (parent, child), weight status homogeneity, parent education, household income, and area-level SES. We also assessed the parent–child PA relationship as measured by questionnaires. When PA was measured via pedometers, we observed a significant relationship between parents’ and children’s PA. Further, this relationship was stronger for higher income families and parents with a graduate degree, however the effects did not reach statistical significance. None of the other variables moderated this relationship. Using questionnaires, a relatively smaller parent–child PA relationship was found.

We found a 260 step/day increase in the children’s steps/day for every 1000 step/day increase in the parents’ steps/day, which was a small to medium sized effect (*r* = .25, *r*_partial_ = .24). Several studies in recent years have assessed the parent–child PA relationship using pedometers in both children and parents and all have observed significant findings with at least one parent [8, 10–13]. For example, significant father-child and mother-child step relationships were observed in a slightly younger sample than ours (aged 4–7 years) in the Czech Republic, however the effect sizes were larger than in our sample [11]. A 2500 step/day increase in mothers’ weekday/weekend day steps was related to an increase of 1143/928 weekday/weekend day steps in the children, and a 2500 step/day increase in fathers’ weekday/weekend day steps resulted in an increase of 903/753 weekday/weekend day step increase in the children. Combined these findings suggest that children and their parents accumulate similar amounts of ambulatory activity throughout the day. Thus, interventions designed to get children moving more throughout the day could be enhanced by including a parent component. Indeed a meta-analysis of family-based interventions found significant but small effects across 19 studies [54].

Our study suggests that the parent–child PA relationship as measured by pedometers does not exhibit a same gender (i.e., gender homogeneity), child gender, or parent gender effect. The literature on gender specific parent–child PA relations using pedometers is quite mixed. For example, significant mother-offspring but not father-offspring step correlations were found in a sample of 8–18 year-old youth in France [10]. Contrarily, fathers’ steps (but not mothers’ steps) was related to the steps of their children in a sample of Spanish children aged 8–9 years and their parents [14]. In contrast, father-son, father-daughter, and mother-son step relationships were observed in a sample of 5–19 year-old Canadian youth and their parents [8]. Yet the relationship between mothers and their daughter’s steps did not reach significance (*p* = .08). Another study found father-daughter, father-son, mother-daughter, and mother-son step relationships on weekends, however the father’s steps was not related to his daughter’s steps on weekdays [13]. A limitation of these studies is that they did not formally test whether the slopes in each groups were significantly different from one another, but rather subjectively compared the size of effects and the significance of the coefficient. Because we formally tested these interactions, our findings provide more conclusive evidence. A study by Jago and colleagues [24] that assessed MVPA using accelerometers, illustrates the importance of formally testing interactions. From an inspection of the beta coefficients, the magnitude of effects of the father-son and father-daughter PA relationships appeared to be similar. For mothers, the effects appeared to be stronger for daughters than sons. A formal test however showed that the mother-child PA relationship was not significantly different for boys and girls.

In addition to testing differences by gender, and guided by the IMPAP, we also explored SES and weight status variables as potential moderators of the parent–child PA relationship. Correlations were higher for parents who had completed graduate school and for those who made > $80,000/year, but the results did not reach statistical significance. Weight status of the child and parent, and gender homogeneity were not effect modifiers. A few studies have examined SES moderators of the parent–child PA relationship. Jacobi and colleagues [10] observed higher correlations between mother-offspring pedometer-determined PA for employed mothers compared to unemployed mothers however the differences were not empirically tested. In a sample of 5–6 year-olds, mothers’ and children’s accelerometer-measure MVPA was related regardless of the mothers’ education. However, fathers’ and children’s MVPA was only significant related for fathers with high education (i.e., had attended university) [19]. To the contrary, Fridlund Dunton and colleagues [55] found that children and parents whose household income was < $30,000/year performed more MVPA together (indicating PA co-participation) than those with a household income >$100,000/year. Similar to our study, Jago and colleagues [9] did not find that parent BMI modified the parent–child PA relationship with 431 parent–child dyads. Taken together, our exploration of several potential moderators provides a unique contribution to the literature and suggests that interventions for families with children aged 7–8 years do not need to be individually tailored by SES, weight status, or gender. Future research should explore if other parental attributes or parenting styles are moderating factors.

A stronger child–parent PA relationship was found when PA was measured objectively using pedometers, compared to when measured subjectively using questionnaires. Similar to our study, a meta-analysis recently reported slightly higher effects when PA was measured using objective *vs* subjective measures, yet the difference was not significant [3]. One explanation is that the higher degree of measurement error that comes with using self- or proxy-report surveys attenuated the effects [6]. Thus, the parent–child PA relationship as measured by pedometers is more precise estimate of this relationship. A second potential explanation is there is stronger familial aggregation for total PA (captured by pedometers) compared to sport and volitional activities which are often captured by questionnaires [8]. In support of this, a significant mother-daughter PA relationship as found for accelerometer-measured counts/min in a sample of 5–12 year old daughters and their mothers, but not for MVPA. Another study however, found non-significant parent-child PA relationships for both counts/min and MVPA [9]. A final consideration is that we measured parent leisure time MVPA during the last seven days and child usual PA. The slight differences in the measures may partially explain the smaller effects found in the questionnaire analysis. Regardless, our results reinforce the importance of using objective measures of PA in both parents and children when possible.

It is important to consider the potential reasons for why active parents have active children. Though this relationship is often described as parental “modeling” or observational learning [4], it is likely due to many factors including genetics, co-activity, and parenting practices and beliefs. It is also possible that children influence their parents [2, 56]. Further, parents could influence their children through several mechanisms including children’s enjoyment, motivation, perceived competence, and/or self-efficacy for PA [2]. A greater understanding of these complex relationships is important for advancing theory in this area. Further, new advances in accelerometry such as GPS tracking and proximity tagging will be useful objective tools for teasing apart when parents and children are being active together, and hence when observational learning is likely occurring.

Strengths of the study include the objective measure of PA used with both parents and children, the large sample size, and the examination of several moderators. Multiple imputation of missing data allowed us to retain a large sample, reduce biases, and to test several potential moderators. But, some limitations should also be acknowledged. First, because the participants were volunteers, and only 39 % of the participants from the baseline phase of the SHAPEs study completed follow-up phase, a self-selection bias could exist. Indeed, the parents in our sample were more educated and higher income earners than the general population in the region. Second, the study is cross-sectional and non-experimental; therefore, we cannot assume that the parents’ *caused* their children to take more steps themselves. To date, no studies have examined the parent–child PA relationship over time using an objective measure of PA in both parents and children. As such, longitudinal and experimental studies are needed to establish temporal precedence and determine if there is evidence of a causal relationship. Third, despite pedometers providing an objective measure of PA, they have limitations. Because the pedometers were unsealed and parents’ recorded theirs and their children’s steps each evening, they could have unknowingly increased their activity or made a recording error. Pedometers are also not able to provide an indication of missing wear time, and thus we had to rely on the logbooks to determine if the monitor was not worn. Further, reactivity could have occurred whereby the parents and children increased their activity level in response to wearing an activity monitor. Several studies have examined the patterns of PA across the week to determine if there is an initial increase in PA and then a leveling off. No evidence of reactivity was found with sealed and unsealed pedometers in both children and adults [36, 57–59]. Two studies have also shown no differences in step counts with sealed and unsealed pedometers in children and adults [60, 61]. In adults, however, evidence of reactivity has been demonstrated when a covert condition (i.e., participants were unaware that their PA is being measured) was compared to a condition where participants were aware their PA was being monitored [62–64]. Finally, we acknowledge the current recommendation for pedometers is seven days of monitoring [65]. When this study was designed, there was no consensus on the number of days of monitoring required to measure habitual PA, and several studies have shown that four days of monitoring provide reliable estimates of habitual PA [21].

## Conclusions

Our findings demonstrate that active parents tend to have active children. Stronger parent–child PA relationships were observed with pedometers compared to questionnaires, which highlights the importance of using objective measures, and may help explain the mixed findings observed in the literature. Interventions designed to get children moving more throughout the day could benefit from including a parent component. Future research should explore the mechanisms by which parents influence their children, and other parent attributes and styles as potential moderators.

## Abbreviations

BMI: Body mass index
IMPAP: Integrated Model of Physical Activity Parenting
METS: Metabolic equivalent
MVPA: Moderate-to-vigorous physical activity
PA: Physical activity
SC: Steps count
SES: Socioeconomic status
SHAPEs: Spatial Health Assessment of Physical Environments

## References

[1] Sallis JF, Prochaska JJ, Taylor WC (2000). A review of correlates of physical activity of children and adolescents. Med Sci Sports Exerc.

[2] Davison KK, Mâsse LC, Timperio A (2013). Physical activity parenting measurement and research: Challenges, explanations, and solutions. Childhood Obes.

[3] Yao CA, Rhodes RE (2015). Parental correlates in child and adolescent physical activity: A meta-analysis. Int J Behav Nutr Phys Act..

[4] Rhodes RE, Quinlan A, Beauchamp M, Eys M (2014). The family as a context for physical activity promotion. Group Dynamics in Exercise and Sport Psychology.

[5] Biddle SJ, Atkin AJ, Cavill N, Foster C (2011). Correlates of physical activity in youth: A review of quantitative systematic reviews. Int Rev Sport Exerc Psychol.

[6] Trost SG, Loprinzi PD (2011). Parental influences on physical activity behavior in children and adolescents: a brief review. Am J Lifestyle Med.

[7] Fuemmeler BF, Anderson CB, Mâsse LC (2011). Parent–child relationship of directly measured physical activity. Int J Behav Nutr Phys Act..

[8] Craig CL, Cameron C, Tudor-Locke C (2013). Relationship between parent and child pedometer-determined physical activity: a sub-study of the CANPLAY surveillance study. Int J Behav Nutr Phys Act.

[9] Jago R, Fox KR, Page AS, Brockman R, Thompson JL (2010). Parent and child physical activity and sedentary time: do active parents foster active children?. BMC Public Health.

[10] Jacobi D, Caille A, Borys J-M (2011). Parent-offspring correlations in pedometer-assessed physical activity. PLoS One.

[11] Sigmund E, Badura P, Vokacova J, Sigmundová D (2016). Parent–child relationship of pedometer-assessed physical activity and proxy-reported screen time in Czech families with preschoolers. Int J Environ Res Publ Health.

[12] Sigmundová D, Sigmund E, Vokáčová J, Kopčáková J (2014). Parent–child associations in pedometer-determined physical activity and sedentary behaviour on weekdays and weekends in random samples of families in the czech republic. Int J Res Publ Health.

[13] Sigmund E, Sigmundová D, Bad'ura P, Vorácová J (2015). Relationship between Czech parent and child pedometer-assessed weekday and weekend physical activity and screen time. Cent Eur J Public Health.

[14] Saavedra J, Escalante Y, Domínguez A, García‐Hermoso A, Hernández‐Mocholí M (2014). Prediction of correlates of daily physical activity in Spanish children aged 8–9 years. Scand J Med Sci Sports.

[15] Freedson PS, Evenson S (1991). Familial aggregation in physical activity. Res Q Exerc Sport.

[16] Moore LL, Lombardi DA, White MJ, Campbell JL, Oliveria SA, Ellison RC (1991). Influence of parents’ physical activity levels on activity levels of young children. J Pediatr.

[17] Barnes AT, Plotnikoff RC, Collins CE, Morgan PJ (2015). Maternal correlates of objectively measured physical activity in girls. Matern Child Health J.

[18] Hesketh KR, Goodfellow L, Ekelund U (2014). Activity levels in mothers and their preschool children. Pediatrics.

[19] Matarma T, Tammelin T, Kulmala J, Koski P, Hurme S, Lagström H. Factors associated with objectively measured physical activity and sedentary time of 5–6-year-old children in the STEPS Study. Early Child Dev Care. 2016:1–11.

[20] Poitras VJ, Gray CE, Borghese MM (2016). Systematic review of the relationships between objectively measured physical activity and health indicators in school-aged children and youth. Appl Phys Nutr Metab.

[21] Tudor-Locke C, McClain JJ, Hart TL, Sisson SB, Washington TL (2009). Pedometry methods for assessing free-living youth. Research Q Exerc Sport.

[22] Tudor-Locke C, Bassett D, Shipe M, McClain J (2011). Pedometry methods for assessing free-living adults. J Phys Act Health..

[23] McNamara E, Hudson Z, Taylor SJ (2010). Measuring activity levels of young people: The validity of pedometers. Br Med Bull.

[24] Jago R, Sebire SJ, Wood L (2014). Associations between objectively assessed child and parental physical activity: A cross-sectional study of families with 5–6 year old children. BMC Public Health.

[25] Fairchild AJ, McQuillin SD (2010). Evaluating mediation and moderation effects in school psychology: A presentation of methods and review of current practice. J Sch Psychol.

[26] Holt NL, Kingsley BC, Tink LN, Scherer J (2011). Benefits and challenges associated with sport participation by children and parents from low-income families. Psychol Sport Exerc.

[27] Humbert ML, Chad KE, Spink KS (2006). Factors that influence physical activity participation among high-and low-SES youth. Qual Health Res.

[28] Thompson J, Jago R, Brockman R, Cartwright K, Page A, Fox K (2010). Physically active families–de‐bunking the myth? A qualitative study of family participation in physical activity. Child Care Health Dev.

[29] Colley RC, Garriguet D, Janssen I, Craig CL, Clarke J, Tremblay MS (2011). Physical activity of Canadian children and youth: Accelerometer results from the 2007 to 2009 Canadian Health Measures Survey. Health Rep.

[30] Colley RC, Garriguet D, Janssen I, Craig CL, Clarke J, Tremblay MS (2011). Physical activity of Canadian adults: accelerometer results from the 2007 to 2009 Canadian Health Measures Survey. Health Rep.

[31] Carson V, Spence JC, Cutumisu N, Boule N, Edwards J (2010). Seasonal variation in physical activity among preschool children in a northern Canadian city. Res Q Exercise Sport.

[32] Carson V, Spence JC, Cutumisu N, Cargill L (2010). Association between neighborhood socioeconomic status and screen time among pre-school children: a cross-sectional study. BMC Public Health.

[33] Pabayo R, Spence JC, Cutumisu N, Casey L, Storey K (2012). Sociodemographic, behavioural and environmental correlates of sweetened beverage consumption among pre-school children. Public Health Nutr.

[34] Spence JC, Carson V, Casey L, Boule N (2011). Examining behavioural susceptibility to obesity among Canadian pre-school children: The role of eating behaviours. Int J Pediatr Obes..

[35] Trost SG, Pate RR, Freedson PS, Sallis JF, Taylor WC (2000). Using objective physical activity measures with youth: How many days of monitoring are needed?. Med Sci Sports Exerc.

[36] Vincent SD, Pangrazi RP (2010). Does reactivity exist in children when measuring activity levels with pedometers?. Pediatric Exerc Sci.

[37] Horvath S, Taylor DG, Marsh JP, Kriellaars DJ (2007). The effect of pedometer position and normal gait asymmetry on step count accuracy. Appl Physiol Nutr Metab.

[38] Schneider P, Crouter S, Lukajic O, Bassett D (2003). Accuracy and reliability of 10 pedometers for measuring steps over a 400-m walk. Med Sci Sports Exerc.

[39] Beets MW, Patton MM, Edwards S (2005). The accuracy of pedometer steps and time during walking in children. Med Sci Sports Exerc.

[40] Telford A, Salmon J, Jolley D, Crawford D (2004). Reliability and validity of physical activity questionnaires for children: The children’s leisure activities study survey (CLASS). Pediatric Exerc Sci.

[41] Godin G, Shephard R (1985). A simple method to assess exercise behavior in the community. Can J Appl Sports Sci.

[42] Jacobs DR, Ainsworth BE, Hartman TJ, Leon AS (1993). A simultaneous evaluation of 10 commonly used physical activity questionnaires. Med Sci Sports Exerc.

[43] DeOnis M, Onyango AW, Borghi E, Siyam A, Nishida C, Siekmann J (2007). Development of a WHO growth reference for school-aged children and adolescents. Bull World Health Organ.

[44] World Health Organization. Global Strategy on Diet, Physical Activity and Health: What is overweight and obesity?; 2015; http://www.who.int/dietphysicalactivity/childhood_what/en/. Accessed 24 Oct 2016.

[45] Carson V, Spence J (2010). Seasonal variation in physical activity among children and adolescents: a review. Pediatric Exerc Sci.

[46] Statistics Canada. 2006 census dictionary; 2009. http://www12.statcan.ca/english/census06/. Accessed 26 May 2016.

[47] DMTI Spatial Inc: GeoPinPoint^TM^ Suite (Version 6.4) [Software]. Markham, Ontario; 2008.

[48] Canada S. 2006 census dictionary. http://www12.statcan.ca/census-recensement/2006/ref/dict/geo021-eng.cfm. Accessed 24 Oct 2016.

[49] Statistics Canada. 2006 census of population, catalogue No. 94-581-XCB2006002. 2006. https://www12.statcan.gc.ca/census-recensement/2006/dp-pd/prof/rel/Ap-eng.cfm?LANG=E&APATH=3&DETAIL=1&DIM=0&FL=A&FREE=0&GC=0&GID=0&GK=0&GRP=0&PID=94534&PRID=0&PTYPE=89103&S=2&SHOWALL=0&SUB=0&Temporal=2006&THEME=81&VID=0&VNAMEE=&VNAMEF=. Accessed 26 May 2016.

[50] Tabachnick BG, Fidell LS (2007). Using multivariate statistics.

[51] Graham JW, Olchowski AE, Gilreath TD (2007). How many imputations are really needed? Some practical clarifications of multiple imputation theory. Prev Sci.

[52] Cohen J (1992). A power primer. Psychol Bull.

[53] Aiken LS, West SG (1991). Multiple regression: Testing and interpreting interactions.

[54] Brown HE, Atkin AJ, Panter J, Wong G, Chinapaw MJ, Sluijs E (2016). Family‐based interventions to increase physical activity in children: a systematic review, meta‐analysis and realist synthesis. Obes Rev.

[55] Fridlund Dunton G, Liao Y, Almanza E (2012). Joint physical activity and sedentary behavior in parent-child pairs. Med Sci Sports Exerc..

[56] Zebedee JA, Gibbons SL, Naylor P-J (2010). Family influence on physical activity: exploring the nature of reciprocal relationships. PHEnex J..

[57] Craig CL, Tudor-Locke C, Cragg S, Cameron C (2010). Process and treatment of pedometer data collection for youth: The Canadian Physical Activity Levels among Youth study. Med Sci Sports Exerc.

[58] Rowe DA, Mahar MT, Raedeke TD, Lore J (2004). Measuring physical activity in children with pedometers: Reliability, reactivity, and replacement of missing data. Pediatr Exerc Sci.

[59] Behrens TK, Dinger MK (2007). Motion sensor reactivity in physically active young adults. Res Q Exerc Sport.

[60] Ozdoba RS, Corbin CB, Lemasurier GC. Does reactivity exist in children when measuring activity levels with unsealed pedometers? Pediatr Exerc Sci. 2004:158–166.

[61] Matevey C, Rogers LQ, Dawson E, Tudor-Locke C (2006). Lack of reactivity during pedometer self-monitoring in adults. Meas Phys Educ Exerc Sci.

[62] Clemes SA, Matchett N, Wane SL (2008). Reactivity: An issue for short-term pedometer studies?. B J Sports Med.

[63] Clemes SA, Parker RA (2009). Increasing our understanding of reactivity to pedometers in adults. Med Sci Sports Exerc.

[64] Clemes SA, Biddle SJ (2013). The use of pedometers for monitoring physical activity in children and adolescents: Measurement considerations. J Phys Act Health.

[65] Lubans DR, Plotnikoff RC, Miller A, Scott JJ, Thompson D, Tudor-Locke C (2015). Using pedometers for measuring and increasing physical activity in children and adolescents The next step. Am J Lifestyle Med.

